# Targeting the Filipino gut microbiota in the management of hypertension

**DOI:** 10.1186/s43044-024-00440-2

**Published:** 2024-01-25

**Authors:** Abraham C. Sianoya, Nemencio A. Nicodemus Jr., Leslie Michelle M. Dalmacio

**Affiliations:** 1 College of Medicine, University of the Philippines Manila, Manila, Philippines; 2 Department of Biochemistry and Molecular Biology, College of Medicine, University of the Philippines Manila, Manila, Philippines

**Keywords:** Gut microbiota, Filipino adults, Hypertension

## Abstract

**Background:**

Hypertension is a major health problem in the Philippines, being the second leading disease and the second leading factor driving the most death and disability in the country. Despite efforts made toward increasing awareness, improving availability of medications, and strengthening patient adherence, more than 7 in every 10 hypertensive Filipinos still have uncontrolled hypertension.

**Main body:**

In the recent years, the role of gut microbiota in hypertension has been highlighted, with studies showing alterations in the gut microbiota of hypertensive individuals and its positive effect on the pharmacokinetics of some antihypertensive drugs.

**Conclusions:**

These findings show how gut microbiota can be an important but possibly overlooked consideration in the management of hypertension in the Philippines. Clinicians might benefit from maximizing the relationship between gut microbiota and hypertension to achieve good BP control and ultimately address the burden of uncontrolled hypertension in the country.

## Background

Hypertension is a major health problem in the Philippines, being the second leading disease [1] and the second leading factor driving the most death and disability in the country [2]. It affects one in every five Filipinos aged 20 years old and above [3]. One study that screened 177, 176 volunteer adults (≥ 18 years) revealed a prevalence of 39%, almost half of whom are on antihypertensive medication [4]. However, only 58% of those on medication had good blood pressure (BP) control or with BP < 140/90 mmHg. Despite efforts made toward increasing awareness, improving availability of medications, and strengthening patient adherence, more than 7 in every 10 hypertensive Filipinos still have uncontrolled hypertension [4], thereby prompting possibly new or recalibrated approaches to other aspects of treatment.

## Main text

In 2013, the potential role of gut microbiota in hypertension was highlighted when a meta-analysis involving 702 hypertensive patients found that probiotic fermented milk significantly reduced systolic and diastolic BP compared with placebo [5], paving the way for succeeding human and animal studies exploring the relationship between gut microbiota and hypertension. In 2019, the first population-based cohort study on such relationship showed that gut microbial diversity was negatively correlated with hypertension while taxa-specific analyses revealed that the genera *Robinsoniella* and *Catabacter* were positively associated with both hypertension and systolic BP [6]. *Akkermansia*, on the other hand, was associated with normotension, while *Sporobacter* and *Anaerovorax* had an inverse relationship with hypertension [6].

The abundance of specific bacterial populations was also observed to be altered in hypertensive humans [7] and in hypertensive models of rats [8], confirming the presence of gut dysbiosis. For instance, in individuals with hypertension, other notable genera seen to have higher abundance include *Alistipes, Desulfovibrio,* and *Klebsiella* while those with lower abundance are *Roseburia, Faecalibacterium, and Ruminococcus* [7]. In animal models of hypertension, a decrease in microbial richness and a marked increase in the Firmicutes/Bacteroidetes ratio were observed [8]. This dysbiosis was associated with a decrease in acetate- and butyrate-producing bacteria and increase in lactate-producing bacterial population. Interestingly, oral minocycline could rebalance such dysbiosis in their rat model of hypertension [8]. In addition, as proof of concept, when feces from hypertensive rats were transplanted into healthy rats, typical hypertension resulted, and when feces from healthy rats were transplanted to hypertensive rats, blood pressure was lowered [9].

Among the first-line antihypertensive drugs given in the Philippines is amlodipine, a calcium channel blocker [10]. When given to antibiotic-treated rats, amlodipine became more bioavailable [11] as was observed for nifedipine [12]. This shows that perturbations in the gut microbiota from antibiotic administration can positively affect the pharmacokinetics of antihypertensive drugs. In turn, other common antihypertensives prescribed in the country such as losartan, captopril, and enalapril have been documented in animal studies to affect the abundance of certain bacterial populations in rats as well as metoprolol in hypertensive humans [13].

## Conclusions

Taken together, the mentioned findings show how gut microbiota can be an important but possibly overlooked consideration in the management of hypertension. While efforts are made at improving patient adherence and access to medications, clinicians in the country might also benefit from maximizing the relationship between gut microbiota and hypertension to achieve good BP control. To achieve this, current progress in gut microbiota research in the Philippines must expand to include not only metabolic disorders such as obesity and diabetes [14] but also hypertension. Future directions for research might include more human studies to validate findings from animal studies and to explore how gut microbiota can be modulated through probiotics, antibiotics, diet, and exercise, to achieve BP control and ultimately address the burden of uncontrolled hypertension in the country.

## Data Availability

Please refer to references

## Abbreviation

BP: Blood pressure
